# Fostering the Reconstruction of Meaning Among the General Population During the COVID-19 Pandemic

**DOI:** 10.3389/fpsyg.2020.567419

**Published:** 2020-10-28

**Authors:** Marco Castiglioni, Nicolo’ Gaj

**Affiliations:** ^1^“R. Massa” Department of Human Sciences, University of Milano-Bicocca, Milan, Italy; ^2^Department of Psychology, Catholic University of the Sacred Heart, Milan, Italy

**Keywords:** COVID-19, psychological distress, meaning construction process, sense of coherence (SOC), limits of the biomedical model, general population, clinical psychology, resilience

## Abstract

The COVID-19 outbreak has seen people in many countries asked to radically modify their way of life in compliance with sweeping safety measures. During the current crisis, technology is turning out to be key, in that it allows practitioners to deliver psychological services to people who would otherwise be unreachable. However, professionals cannot solely rely on their traditional modes of practice, in that different methods are required to bring to light the needs of those affected by the emergency. People are being overwhelmed by a cascade of unusual and unexpected events that are putting a strain on their everyday routines and usual meaning-making systems; ongoing challenges to their employment and financial status will likely divert personal resources away from psychological well-being. We therefore argue that psychologists should also consider the needs of the general population. Among those who may require help–aside from the main targets of psychological intervention, such as healthcare personnel and COVID-19 patients and their relatives–specific attention should be paid to those who are not at the center of the crisis. We suggest that this large segment of potential users may benefit from a non-medical approach focused on the promotion of meaning-making processes. Indeed, the disruptive nature of the current situation hinders sense-making and threatens to undermine psychological balance and well-being, at an individual as well as at a societal level. The present article proposes a methodological perspective based on the reconstruction of meaning-making processes (sense of coherence, predictability, metaphors, narratives). Specifically, psychological interventions should promote personal and collective resources with a view to: “normalizing” current distressful experiences (i.e., acknowledging that such reactions are normal in light of the present situation); widening the observational field, taking relational contexts into account, and promoting an understanding of distressful experiences as coping strategies; fostering meaning-making/reconstruction processes through the use of appropriate metaphors and narratives; promoting a sense of coherence. We present two clinical vignettes to illustrate how these principles might be applied in practice. In conclusion, the exceptional psychological challenges posed by the COVID-19 pandemic require practitioners to adopt a broad and flexible perspective on clinical intervention.

## Introduction

The recent coronavirus disease (COVID-19) outbreak has seen people in many countries asked to radically modify their everyday behaviors in compliance with sweeping safety measures introduced by European, Asian, and American governments. It is well documented that having to cope with infectious outbreaks places a considerable strain on people’s lives^1^ (see Brooks et al., 2020; Pfefferbaum and North, 2020). During the COVID-19 crisis, entire populations have suddenly found themselves struggling with an invisible enemy that can potentially strike anyone and may only be confronted by forgoing–although temporarily–the company of significant others and enduring severe reductions in living space and personal freedoms.

In view of the above, it is our opinion that scholars should extend their research focus beyond those “in the frontline,” such as healthcare personnel, COVID-19 patients, and their relatives, to investigate the impact of the emergency on the public at large, given that “emotional distress is ubiquitous in [populations affected by public health emergencies]–a finding certain to be echoed in populations affected by the COVID-19 pandemic” (Pfefferbaum and North, 2020, p. 1). Indeed, recent works have pointed up the need to study the “psychological effects of the COVID-19 pandemic across the whole population and in specific vulnerable groups” (Holmes et al., 2020, p. 10) in light of the clearly adverse effects of quarantining on psychological well-being (Brooks et al., 2020) and long-lasting stressors related to the outbreak (Sood, 2020).

The negative effects of forced and prolonged mass quarantining include boredom, loneliness, social disconnectedness, a sense of lack of meaning, relationship breakdowns, anger, avoidance behaviors, unhealthy behaviors, and abnormal emotional reactions (Brooks et al., 2020; Holmes et al., 2020; Pfefferbaum and North, 2020). The unavailability of routine assistance due to the closure of businesses and institutions can further amplify the impact of the crisis, potentially affecting large sectors of the population: consider, for example, families with schoolchildren, persons living alone, elderly persons and their caregivers, unemployed persons, those on a low income, people in unstable social conditions, homeless persons, and other vulnerable categories (see World Health Organization, 2020). It is also likely that the pandemic will have enduring psychological consequences on an unprecedentedly global scale in the later stages of mass home confinement (when freedom of movement has been at least partially restored) and over the long-term aftermath of the lockdown. A recent survey by the American Psychological Association (2020) revealed that the pandemic has altered all aspects of personal and family life, from health and work to education and exercise. Parents of children under 18 are among the categories most affected by pandemic-related stress. The emergency has upset daily routines (during both the initial lockdown and the ongoing phase of gradual resumption of normal activity),^2^ severely impinging upon personal as well as family and interpersonal projects and drastically modifying habitual modes of interaction at both the familial and social levels.

The nature of the disease itself prompts significant uncertainty about the future. People’s concerns can be many and varied, spanning fears for their occupational and financial status during the recovery phase; anxiety surrounding new behaviors that the epidemiological situation may require them to adopt in the interests of their personal safety (such as novel modes of interacting with strangers, alternative workplace procedures or leisure-time activities, etc.); worry that a new wave of disease may hit the world, or that we may have to live with the virus for a long time to come. It is likely that such sources of distress will wield a profound effect on community mental health, although not necessarily in terms of diagnosable disorders.

Policy makers and psychological scientists thus need to be keenly aware of the wide-ranging psychological impact of the pandemic if they are to design targeted psychological surveillance and intervention strategies for helping people to cope with it (see Higgins, 2020).

Accordingly, this paper presents a possible perspective on addressing COVID-induced forms of distress–which are often subtle and elusive, but, nevertheless, worthy of attention–in people who are not at the center of the crisis, that is to say, those impacted by safety measures but not necessarily directly affected by COVID-19. First, in the “Critical Issues for the Delivery of Psychological Assistance During the COVID-19 Outbreak” section, we acknowledge the key role of technology in providing access to, or ensuring the continuity of, psychological services to this population. We also flag conceptual issues with interventions–whether technologically mediated or face-to-face–that are strongly rooted in the biomedical model and draw on a symptom-centered approach. While recognizing the importance of taking symptoms into account in all clinical intervention contexts, we argue that symptom-centered approaches may be of limited benefit when applied to forms of distress whose diagnostic status is unclear (or inexistent).

We therefore suggest that a broader perspective on psychological intervention is needed. In the “Making Sense of COVID-19: Routine, Predictability, Narrative, Sense of Coherence, and Resilience” section, we explore the link between COVID-19-related distress and the disruption of meaning, going on to outline key theoretical aspects of the meaning-making process, such as routine, predictability, narratives, sense of coherence, and resilience. Then, in the “Enhancing Processes of Meaning Making About COVID-19 in the General Population” section, we describe a multipronged perspective on psychological intervention, whose main features are coherent communication strategies, the de-pathologization of distressful reactions to the pandemic, a widening of the field of observation from individuals to their relational contexts, and the negotiation of suitable personalized metaphors for enhancing psychological well-being. In the “Clinical Vignettes” section, we present two clinical vignettes to practically illustrate how our proposed perspective might be applied in practice. Finally, in the “Concluding Remarks” section, we conclude by summarizing the broad criteria that we believe should inform intervention targeting COVID-19-induced psychological issues.

## Critical Issues for the Delivery of Psychological Assistance During the COVID-19 Outbreak

The situation in countries that have implemented mass home confinement in response to the COVID-19 outbreak raises serious mental health concerns. However, policy makers dealing with large-scale public health challenges typically overlook recommendations to supplement physical health interventions with mental health programs (Perrin et al., 2009). Due to the perception that physical assistance is more crucial or urgent, and the awareness that enormous financial, political, and even communication resources are required to address the epidemiological aspects of pandemics, the psychological impact of the emergency may only receive attention at a later stage. Clearly, this has major implications for people’s mental health and the availability of psychological assistance, compounding the critical factors that already routinely hinder access to mental health services.

Under normal conditions, a high proportion of people suffering from mental health problems do not receive care. It is well documented that the barriers to seeking treatment are attitudinal rather than structural. In other words, people are more frequently hindered from looking for help by their own thoughts and beliefs than by practical obstacles to accessing psychological services (such as a lack of financial resources, transportation, or availability). In a World Health Organization (WHO) study conducted by Andrade et al. (2014) in 24 countries, people with mental health issues reported not seeking treatment for two main reasons: 1. They wished to handle the problem independently. 2. Their self-perceived need for treatment was low, associated with the expectation that the problem would get better on its own. Now, in a pandemic scenario where the greatest emphasis is understandably laid on physical protection and the implementation of effective safety measures, it is likely that such attitudinal barriers may impact even further on people’s willingness to ask for psychological assistance, at least in the initial stages of the emergency. We further hypothesize that a crisis like the COVID-19 pandemic will lead individuals to increasingly focus on ongoing challenges to their employment and financial status, and that this can easily lead them to divert personal resources away from caring for their mental health. Such a dynamic may be exacerbated by two concurrent factors. First, media and government communications about the risks associated with the pandemic and the safety measures to be adopted are frequently perceived as inadequate, alarming, and even contradictory (see Brooks et al., 2020), while the style, approach, and content of media and government communications can accentuate perceptions that some issues are more urgent than others, thus influencing the focus of people’s attention. Second, growing recourse to drugs for stress-related symptoms may favor the adoption of shallow solutions to complex problems (Ao, 2020; Pesce, 2020). Furthermore, while the sample in the above-cited study by Andrade and colleagues was assessed using a DSM-IV-based diagnostic approach, in the current pandemic, people may suffer from forms of distress that are not strictly ascribable to traditional psychopathology or that fall below the diagnostic threshold and are therefore less immediately recognizable. Hence, during a pandemic, a lack of psychological surveillance is likely to become a major problem due to the combined effects of all these factors. There is a severe risk that people’s psychological needs will remain unexpressed and untreated.

In a scenario like the present emergency, the use of technology to provide psychological assistance is turning out to be key, as the only viable way to overcome the necessary barrier of obligatory physical distancing. This last-mentioned concept has very recently replaced that of social distancing (Gunnell et al., 2020), in acknowledgment of the distinction between social connections and physical connections, and reflecting the fact that the former may be ensured by technological devices, while the latter are generally precluded during large-scale sheltering in place.

Although it is beyond the scope of this article to analyze in depth the use of technological devices for psychological purposes, we should note the undoubted advantages of deploying technology during an infectious outbreak. First, research has documented the efficacy of mental health services mediated by technological devices^3^ in targeting psychiatric disorders across a range of populations and settings (Bashshur et al., 2016; Hubley et al., 2016; Ebert et al., 2018; Shore et al., 2020). Second, the deployment of technology is generally accepted and positively evaluated by clients (Bashshur et al., 2016; Hubley et al., 2016). Third, through the use of technological devices, psychological assistance can be made virtually available to all those who need it, including persons living in isolated regions or those whose liberty of movement is most severely restricted. Fourth, technology-based intervention is financially advantageous for clients, practitioners, and healthcare institutions alike (see Bashshur et al., 2016). Finally, technological mediation may mitigate the shame and stigma associated with attending psychological services, thus facilitating access to mental health assistance (Ebert et al., 2018). In sum, the psychological community has a duty to avail of technology to efficaciously deliver psychological interventions to the population at large. However, despite the huge potential offered by the online medium, certain limitations of most of the technologically mediated psychological services currently on offer should be noted. As Bashshur et al. (2016), as well as Ebert et al. (2018), have pointed out, mediated services are generally designed and delivered in keeping with standard cognitive behavior therapy (CBT) principles. In other words, due to the features of technological devices and the type of interaction that they typically support, these services most frequently target changes in specific behaviors and thoughts and are therefore particularly suited to treating specific psychopathological conditions such as, for example, panic disorders, and PTSD (Bashshur et al., 2016; Hubley et al., 2016). However, telepsychology is a multifaceted and evolving area comprising multiple kinds of services with diverse features. To simplify somewhat, one main type of intervention is closely modeled on face-to-face interaction: the practitioner/client relationship (whether in the area of counseling, education, psychotherapy, supportive intervention, etc.) is merely transferred to a different (technological) medium. Another group comprises more focused interventions, including interactive self-help lessons, virtual or augmented reality exposure-based techniques, serious games, avatar-led sessions, and others.

Both types of intervention–whether or not directly based on CBT principles–tend to be symptom centered, that is to say, they target discrete, well-defined symptoms,^4^ using current diagnostic systems such as the DMS-5 (American Psychiatric Association, 2013) and the ICD-11 (World Health Organization, 2018).^5^ There is ample evidence that this approach is rooted in a biomedical model of clinical intervention that has influenced clinical psychology more profoundly than is commonly believed (Henriques, 2002; Deacon, 2013; Frances, 2013; Castiglioni and Laudisa, 2015). Analogously to its treatment of bodily ailments, the biomedical model conceptualizes forms of mental distress as diseases, that is to say, as clusters of interconnected and concurrent symptoms (with diagnostic status) that form a single framework of disease (Hucklenbroich, 2017). It consequently emphasizes the development and delivery of disorder-specific treatments (Deacon, 2013), such as manualized interventions, behavior protocols, and skills training.

Now, what aspects of the biomedical model might turn out to be problematic when addressing the distress caused by the COVID-19 pandemic and its direct and indirect consequences? In answer, let us focus on two conceptual features of this model.

First, as stated above, it presupposes a sort of isomorphism between bodily problems and mental problems. Mental health issues are understood as forms of dysfunction, that is to say, as problematic (i.e., prejudicial) deviations from normal, physiological functioning. Thus, mental symptoms are viewed as signals that something is going wrong inside the individual: sources of distress are viewed as endogenous, while external factors only influence the expression of inner causes (see Slife et al., 2017). This perspective strongly favors biological explanations for mental health issues (Henriques, 2002; Deacon, 2013; Johnstone and Boyle, 2018).

Second, the biomedical model frames mental problems as relatively context independent, where context is understood as the social, situational, relational, and local conditions or circumstances in which a particular phenomenon occurs (see VandenBos, 2015). In principle, from a biomedical perspective, the diagnostic process may be conceptualized in two ways: as a part-whole explanation, whereby all symptoms are manifestations or components of the disease, or as a causal explanation, whereby all symptoms of a disease are connected by a causal chain (Hucklenbroich, 2017). In both cases, the conceptual resolution, although different in degree, is narrower in focus, centering on a sort of *micro-context* whose elements are internal to the individual (such as personality traits, reinforcement histories, cognitions, etc.) and proximal to the cluster of symptoms (hence generally biological in nature) (see Slife et al., 2017). This is because if symptoms are envisaged as impersonal displays of a discrete disease (see Hucklenbroich, 2017), understanding and diagnosing mental health issues will be seen as relatively independent of other specific situations or circumstances pertaining to the broader context where these issues have arisen (see Bradford, 2010; Jacobs and Cohen, 2010). This *meso*/*macro-context*, whose elements are distal to the cluster of symptoms and interpersonal in nature, thus becomes marginal to understanding an individual’s distress. In sum, such an approach “assigns a secondary role to the social world as a source of ‘triggers’ or ‘stressors’ and offers a particular construction of the person, often as biologically different and vulnerable (…)” (Johnstone and Boyle, 2018, p. 90).

Hence, symptom-centered approaches grounded in the biomedical model bear methodological as well as practical implications when addressing mental health issues arising due to infectious outbreaks. On the one hand, they fail to acknowledge that forms of distress they label as mental problems might be better understood as coping strategies–although painful and onerous in terms of emotional resources–deployed to face extraordinary environmental circumstances (Bentall, 2009; Herman, 2015). They therefore risk overlooking or underestimating the active stance that an individual must adopt in order to cope responsibly with pandemic-related issues. On the other hand, symptom-centered approaches focus heavily on the disability associated with the problem under scrutiny, that is, the impairment interfering with the individual’s ability to function in one or more key life domains (VandenBos, 2015). This emphasis may hinder the development and leverage of personal, familial, and social resources for dealing with distressing experiences; on the contrary, it may amplify the shame, guilt, and stigma often experienced in relation to mental health problems (Bentall, 2009; Deacon, 2013).

This said, a caveat is in order here. We are not suggesting that symptom-centered approaches are to be rejected. We acknowledge that the treatment of symptoms is a key component of all clinical interventions targeting specific manifestations of distress. Furthermore, symptoms may be conceptualized as the “tip of the iceberg,” so to speak, in that they often offer access to a client’s needs and system of meanings. Indeed, symptoms are frequently the reason individuals decide to request a clinical consultation in the first place, as well as the conduit linking the client’s needs with the clinician’s competence. Hence, a careful clinical assessment of symptoms is a key component of any psychological intervention, regardless of the practitioner’s clinical orientation.

Rather, we contend that: 1. Symptom-centered techniques (whether technologically mediated or not) may prove inadequate when used as stand-alone interventions, encouraging a mechanistic reading of complaints and treatment that is exclusively aimed at bringing about the remission of symptoms. 2. They may be unhelpful in cases of milder and/or blurred symptomatology, or manifestations of distress that do not fully meet any given set of diagnostic criteria (see Deacon, 2013). These considerations are relevant to the COVID-19 emergency insofar as we may reasonably assume that the most common sources of distress during infectious outbreaks do not necessarily give rise to classical mental health conditions, in terms of either intensity of distress (i.e., milder and/or subtle forms of distress may be the norm) or quality of subjective experience (i.e., they will likely generate new ways of coping with novel stressors).

We therefore argue that the marked influence of the biomedical model on most psychological practices–including those that are technologically mediated–risks undermining psychological responses to the challenges posed by the COVID-19 outbreak to the population at large.

## Making Sense of COVID-19: Routine, Predictability, Narrative, Sense of Coherence, and Resilience

As described above, the COVID-19 pandemic is generating a set of long-lasting triggers that are highly disruptive of the processes by which people usually make sense of their lives. It poses serious challenges, at a variety of levels, to the systems we all use (both individually and socially) to construct a meaningful sense of the world we live in and even of ourselves. Within the constructivist paradigm^6^ (Neimeyer and Mahoney, 1995; Raskin and Bridges, 2002; Neimeyer, 2009), meaning construction is seen as the most central factor in human psychological life, a key to understanding both typical and atypical mental functioning.

*Meaning making* is a complex and multilayered process that includes cultural, linguistic, social, familial, and psychological (cognitive and emotional) components, among others. Together, these elements give rise to a *lived*, *dynamic sense of intentionality* and *selfhood*. Given the nature and variety of its constituents, meaning is never entirely individually constructed: it is something that we both “make and find” (Shotter, 1993, p. 77). In the specific domain of mental suffering, Guidano (1991, pp. 56–60) defined psychopathology as a “science of meaning,” proposing that “personal meaning organization” shapes the meaning-making process that undergirds the development of self, lending coherence and stability to personal identity (citation omitted for anonymous peer review, Castiglioni et al., 2014). Hence, most forms of mental distress derive from a disruption (or interruption) to meaning making.^7^ For example, in depression (one of the consequences of the COVID-19 pandemic viewed as most likely and harmful by WHO), “depressed persons often report that *they feel disconnected from the world*, that it appears as an *empty place deprived of all meaning*” (Jacobs, 2013, p. 2, italics added).

As long documented in the literature (Kelly, 1955; Bruner, 1990, 1991), there is a close (psycho)logical link between routine, predictability, and meaning. The disruption of everyday routines renders the world unpredictable, thus interfering with sense making, a process that according to Kelly (1955) relies on the ability to “predict and control events.” In circumstances such as these, *time*–one of the core dimensions of human life–is deeply impacted: the *present* is “suspended” (a phenomenon that applies particularly to the confinement phase of managing the pandemic); the *past* no longer provides the wherewithal to interpret the current situation; the *future* becomes unpredictable, and all capacity for forward planning is arrested, given that most aspects of everyday reality have become fuzzy and unrecognizable, causing disorientation and the inability to act (a phenomenon that is typical of the current recovery phase).

The multifaceted process of meaning making described above organizes and expresses itself through metaphors and narratives. *Metaphors* serve to provide us with an idea of something that is hitherto unknown to us by associating it with an object we already know (Battistelli, 2020), a function that bears similarities to the process of “anchoring” described by Moscovici et al. (2001) in his theory of social representations.^8^

*Narratives* are stories, based on metaphors, that convey the meaning of events in our lives over time (Polkinghorne, 2004; Squire et al., 2014); they thus offer a useful guide to interpreting and dealing with unfamiliar phenomena. “Narrative is a form of discourse that links events together across time, and thus, it can display the temporal dimension of human existence. Narrative form captures the notion that human lives are ‘becomings’ or journeys in which actions and happenings occur before, after, and at the same time as other actions and happenings” (Polkinghorne, 2004, p. 58).

Due to their discursive nature, narratives are constructed in and through social interaction. Neimeyer and Sands (2011) described how people organize the “seamless flow of life events,” including negative happenings, into meaningful episodes that reflect personally significant themes, and then seek validation for this framework of meaning in the course of relating to others. From a narrative point of view, individuals construct a life story that is uniquely their own, yet unavoidably shaped by the social discourse of their specific cultural context.

The ability to construct meaningful and consistent narratives that illuminate and explain what is happening, at the individual, familial, and societal levels, appears to be a fundamental (pre)requisite for coping with any situation we may be presented with. Narratives, metaphors, and the set of meanings they convey, all contribute to establishing our *sense of coherence*.

The construct of sense of coherence (SOC), first proposed by Antonovsky (1993, 1987, 1979), offers a helpful framework for integrating personal and contextual factors. SOC is a stable universal construct that applies to all genders, social classes, and cultures (Sagy and Antonovsky, 2000; Eriksson and Lindström, 2005). It may be defined as a global predisposition reflecting the degree to which an individual feels pervasively, lastingly, and dynamically confident that internal and environmental stimuli are structured, predictable, and explainable (*comprehensibility*); that resources are available to meet the demands posed by these stimuli (*manageability*); and that such demands are challenges meriting investment and engagement (*meaningfulness*). Accordingly, sense of coherence would appear to be a key factor in our ability to deal with traumatic events such as the COVID-19 emergency and its direct and indirect aftereffects (Kimhi et al., 2010; Veronese et al., 2013; citation omitted for anonymous peer review).

There is no doubt that the COVID-19 pandemic, which is unprecedented in recent Western history, is a quintessential instance of the “unknown”: one that defies all our usual sense-making systems, challenging our “sense of coherence.” It thus requires the construction of new meanings, as well as of new routines, metaphors, and narratives, or–at the very least–a major revisiting or re-adaptation of existing ones.

As expressed by Prime et al. (2020, p. 9) in a “hot-off-the-press” paper on family well-being during COVID-19, “in recent weeks, families have encountered social disruption; family illness; and, for many, death and grief. They will experience the highest levels of adaptation when they are able to ‘make sense’ of the disaster by incorporating the events into their existing worldview, or by modifying their views, in a way that promotes health, togetherness, and a sense of coherence.”

Significantly, Prime and colleagues view the *family* as playing a key role in the process of meaning (re)construction, given its status as the crucial point of intersection between the individual and social dimensions. They propose a conceptual framework based on systemic and ecological models of human development and family functioning, in which they link the social disruption (public health emergency, financial turmoil, job losses) caused by COVID-19 to family–and particularly child–adjustment, through a cascading process involving family caregivers, family (sub)systems, and family processes (Prime et al., 2020). A key construct in their work is “family resilience,” a construct first developed by Walsh (1998, 2015), who–in a critique of the individualistic “deficit model”–reinterpreted the concept of resilience (traditionally viewed as an exclusively individual trait) according to insights drawn from systemic family therapy. “Walsh (1998, 2015) seminal work on family belief systems in fostering resilience highlights three critical areas in which family beliefs will be implicated in the response to COVID-19: (a) meaning making of adversity, (b) fostering a positive outlook, and (c) transcendence and spirituality” (Prime et al., 2020, p. 9).

Despite its novel status, COVID-19 presents some similarities to other traumatic events such as natural disasters, catastrophes, terrorist attacks, wars, and traumatic loss (Prime et al., 2020), all situations that elicit major distress (Veronese et al., 2010) found that, in a context of ongoing warfare and protracted political violence, the capacity to attribute sense and coherence to uncertainty promoted a sense of efficacy and power. Hence, the ability to construct meaning in uncertain and traumatic conditions may be expected to enhance psychological well-being and quality of life, mitigating the direct and indirect effects of trauma caused by adverse conditions. Furthermore, clinical theory on traumatic loss (Hooghe et al., 2012; Neimeyer et al., 2014; Procaccia et al., 2018 citation omitted for anonymous peer review) suggests that family systems and their crucial role in meaning-making processes contribute to a more advanced ability to make sense of adverse events.

The study of narratives can further our understanding of the processes through which family members maintain or construct a sense of resilience following a shared loss or, as in the case of the current pandemic, a prolonged adverse situation. This is borne out by the finding that mourners who succeeded in gradually integrating their bereavement into their meaning systems report fewer symptoms of complicated grief over the longer term (citation omitted for anonymous peer review; Holland et al., 2010; Procaccia et al., 2018).

Naturally, all of the factors in meaning making just outlined can be influenced, and potentially significantly reinforced, by the use of technological media. As observed in the “Critical Issues for the Delivery of Psychological Assistance During the COVID-19 Outbreak” section, new digital technologies can facilitate the work done in clinical settings; more generally, they can enhance people’s everyday interaction with others. For example, they can enable routines to be maintained that might otherwise be interrupted due to physical distancing requirements, or they can serve to convey and amplify narratives and metaphors, bringing them to the attention of wide audiences, and they can even offer a “laboratory” for the creation of new meanings.

In light of this background, how may the key coping factors we have just described be enhanced to equip the broader population for dealing with COVID-19?

## Enhancing Processes of Meaning Making About COVID-19 in the General Population

One of the first areas requiring intervention is *communication strategy*. As argued by many (Brooks et al., 2020; Holmes et al., 2020), in a situation like the COVID-19 pandemic, all official and media communications should be clear, consistent, and brief, especially those concerning the practical rules to be followed in the interest of avoiding infection and accelerating recovery from the public health and economic crises. In practice, conflicting messages delivered by media and government agencies in many countries have posed an additional challenge to the sense of coherence of the general population, especially in terms of the perceived *comprehensibility* and *manageability* of the pandemic (Antonovsky, 1996; citation omitted for anonymous peer review, Veronese et al., 2013). With regard to comprehensibility, inconsistent information about the nature of COVID-19 undermines the perception that the phenomenon is predictable and explainable. With regard to manageability, confusing communications have prompted serious doubts about the technical and practical resources available to address the pandemic and its consequences.

Second, clinical intervention should foster the *normalization* of psychological reactions to the pandemic. It seems reasonable that–up to a certain degree–fear, anxiety, depressive mood, sorrow, preoccupation, disappointment, insomnia, lessened or increased appetite, inability to concentrate, and many other distressful reactions should be viewed as natural responses to the exceptional triggers generated by the COVID-19 emergency. As argued by Horwitz and Wakefield (2007, p. 9) in relation to depressive symptoms arising in response to adverse events, “such reactions, even when quite intense due to the severity of the experience, are surely part of normal human nature.” It is therefore difficult to justify the current overreadiness to pathologize many “negative” reactions to detrimental stimuli (see Schimmenti et al., 2020; Venuleo et al., 2020). Of course, individuals can react very differently, in terms of both intensity and type of response, depending on a multitude of factors, including previous mental disorders, disadvantaged circumstances, economic difficulties, membership of discriminated-against minority groups, and so on (Johnstone and Boyle, 2018; Prime et al., 2020), and some reactions may be perceived as more “appropriate” than others with respect to society’s standards of “normal.” In any case, we, here wish to flag the risk of what Frances (2013) has termed diagnostic hyperinflation, a phenomenon that entails the medicalization of *ordinary* life. In the context of the current situation, an increase in distress and requests for psychological support should be generally viewed as an appropriate and proportional response to *extraordinary* circumstances that have altered virtually all aspects of everyday life. Thus, an uptick in demand for psychological services might even be seen as desirable, to the extent that it likely reflects self-awareness on the part of those undergoing distress. At the same time, initial psychological assistance should be characterized by *active listening*, with a view to allowing the client’s needs to emerge and be expressed. As argued elsewhere (citation omitted for anonymous peer review, Castiglioni and Laudisa, 2015) in relation to depressive symptoms, throughout the diagnostic process, practitioners should take careful account of the traditional distinction between “endogenous” syndromes, arising in the absence of any apparent external reason, and “reactive” ones, triggered by negative external circumstances.

A third aspect, which is closely interconnected with the second, concerns the crucial importance of *widening the observational field* from individuals to their familial and social–relational contexts (Watzlawick et al., 1967; Bateson, 1972) if we are to fully understand the origins and nature of clients’ suffering and contribute to the effective resolution of their difficulties. As argued above, the biomedical model tends to be a-contextual, limiting its observational (and diagnostic) focus to the individual. This is due to its implicit correlation with the so-called “deficit model.” The latter posits that “psychopathology is the result of dysfunction and distress, which are attributed to some deficiency within the individual. Thus, the onus is on the individual to enact certain changes to reduce distress and dysfunction and consequently improve mental health” (Anglin and Polanco-Roman, 2017, p. 998). In contrast with this predominantly “illness-oriented” biomedical-deficit perspective, more health-oriented paradigms (Antonovsky, 1996; Seligman et al., 2005) emphasize the importance of promoting well-being by focusing both on individuals’ personal strengths and on the resources available to them in their contextual environments, such as the earlier-cited factor of family resilience. The outcomes of previous research on war settings by one of the present authors (citations omitted for anonymous peer review, Veronese et al., 2010, 2012, 2013) suggest that clinical intervention should be designed to reinforce components of positive functioning rather than to “rectify” behaviors, patterns of thinking, or emotions perceived as maladaptive. In keeping with the methodological principle of widening the field of observation, clinical efforts should be directed at increasing clients’ “social capital,” in terms of maximizing and leveraging cohesion at the levels of family, networks of friends, and community. Used to this end, participatory frameworks and action research models can facilitate and enhance therapeutic interventions focused on symptoms (Razer et al., 2009; Smith and Romero, 2010). Overall, the emphasis should be on fostering well-being and strengthening positive coping factors, with a view to obtaining stronger outcomes in uncertain situations without short-term solutions (Nguyen-Gillham et al., 2008; Hunt, 2010).

Group intervention offers a valuable means of targeting this goal. We suggest that the formation of *ad hoc* peer groups–whose meetings could be physical and/or technologically mediated and would be led by a specialized psychologist, at least in the earlier stages–may be key in this regard. Such groups–by sharing their emotional experiences surrounding the pandemic and activating the members’ personal resources–would counteract the sense of meaninglessness and disconnectedness induced by the decrease in the quality and quantity of interpersonal interactions imposed by the COVID-19 emergency. Indeed, the very fact of taking part in a group promotes a sense of being connected to others, which is crucial in the current scenario where individual behaviors are interdependent (in terms of the mutual observance of safety measures), yet people have far fewer opportunities to interact with one another (due to lockdown arrangements and physical distancing rules). Among their intrinsic therapeutic factors, groups can inspire hope, given that they “invariably contain individuals who are at different points along a coping-collapse continuum” (Yalom and Leszcz, 2005, p. 5): hence, mutual interaction among group members may itself instill the hope of future improvements. Furthermore, groups may foster a normalizing outlook on the personal issues and distressful experiences generated by the pandemic: the discovery that many issues and experiences are shared with others could prompt the members of the group to broaden their definition of “normal” (for examples of group interventions fostering meaning-making processes, see Breitbart et al., 2010; Lund et al., 2017). A particularly interesting format recently developed in the context of the COVID-19 pandemic is W I I Thrive,^9^ a group-centered approach aimed at enhancing human dignity and well-being with integrity. Within this framework, individuals are encouraged to form peer groups with a view to connecting with and supporting each other and discussing the challenges of adaptive living, on the basis of a shared set of ideas, values, and practices (G. R. Henriques, personal communication, 28 May 2020).

Finally, spirituality, in terms of either religious beliefs and rituals or a sense of “connectedness” to nature, art, beauty, etc., has been identified as an additional key resource for coping, healing, and fostering personal, familial, and group resilience (Delgado, 2007; Walsh, 2010; Prime et al., 2020). In brief, active social and personal engagement with spiritual and/or religious experiences may contribute to maintaining an adequate sense of coherence, and thus to coping better with psychological distress caused by the COVID-19 pandemic and the resulting socioeconomic crisis (Drageset et al., 2008).

Fourth, interventions aimed at restoring and fostering well-being should focus on processes of *meaning construction*, drawing on *metaphors*, and *narratives* that orient clients in coping with the pandemic and its long-term consequences. Let us consider the “metaphor of war” as an example of this kind of meaning-making process, bearing in mind that the performative power of communication and language enables us to “do things with words” (Austin, 1962).

Throughout this paper, we have more than once drawn a parallel between how people may be affected by the COVID-19 pandemic and the impact on individuals and communities of ongoing armed conflict. It is not by chance that one of the figures most widely used in the media (as well as in everyday conversations among people) to describe the current health emergency has been the war metaphor: we are engaged in a war against an “invisible enemy” (the virus), a war that has its own soldiers, prisoners, heroes, martyrs, traitors, victims, and fallen. Vedovelli (in Milesi, 2020) has critiqued the pervasive use of this metaphorical device, arguing that it risks generating a climate of suspicion, closures, barriers, and clashes. On the contrary, Battistelli (2020), while recognizing the potential insidiousness of the “pandemic as a war” metaphor, has pointed to its effectiveness in conveying a notion of serious threat to people’s safety, health, and lives, thus spurring us to join forces in combatting the disease. In Vedovelli’s view, social communications should be aimed, on the one hand, at making the “inexpressible” (the invisible enemy) understandable through the *logos* of scientific discourse and, on the other, at generating and supporting social relations. The debate between these two scholars illustrates how different metaphors give rise to different narratives and sets of meanings, potentially orienting the way in which we experience, interpret, and deal with the pandemic.

Of course, in our opinion, other metaphors could be viable, too. For example, the metaphor of the journey (Polkinghorne, 2004), which portrays us as currently traveling over very rough terrain with no reliable map, or the bereavement metaphor (Neimeyer et al., 2011), whereby, as a community, we have joined together to mourn our previous way of life, hoping that a new alternative will soon emerge, or the “man-as-scientist” metaphor (Kelly, 1955), according to which we all strive to predict and gain control of events, for example, by researching the causes of this unprecedented phenomenon and studying new solutions for overcoming or managing it. However, the range of metaphors available in a certain cultural context are not infinite. Given that meaning-making processes are embedded in their social–cultural and historical contexts, to some extent, “the affective interpretation of the insurgence of the pandemic scenario, rather than depending on the inherent characteristics of COVID-19, reflects the patterns of affective meanings grounding the cultural milieu at the moment in which the interpretation was enacted” (Venuleo et al., 2020, p. 124).

All these metaphors produce different narratives and interpretations, which in turn may prompt different attitudes and behaviors. In the context of clinical intervention, the practitioner can negotiate suitable personalized metaphors with the client.

## Clinical Vignettes

We now present two clinical vignettes with a view to practically illustrating–although in brief–some aspects of the clinical perspective outlined in the last section.

The first vignette thematizes the normalization of distressful experiences generated by the COVID-19 pandemic and the value of widening the field of observation to include the client’s present and past contextual environment.

C., an only child, is a 49-year-old married woman with two children, one of whom displays difficult personality traits. She lives with her family in the suburbs of Milan. At the age of nine, she lost her father, a farmer, and spent her youth with her mother, a woman – then in her late forties – without a formal education. At the age of 20, she met her husband, whom she describes as “a strong man you can rely on.” However, she has somehow always felt inferior to him: she still believes that she was lucky he “chose her, a simple country girl.” She is now employed by a state-owned enterprise.
During the COVID-19 home confinement, she discovered that she had been in contact with an elderly woman who later passed away due to coronavirus. As required by governmental regulations, C. reached out to the local health authority to report what had happened. She was very worried about her own health status and tried to gather information about the possible consequences of infection. She relayed that the health professionals she spoke with – first by phone and later in person – had displayed a highly detached attitude. In their interactions with her, they focused solely on what had happened during the brief contacts C. had had with the deceased woman and on the measures to be taken as a result. C. was clearly distressed by the situation but had the impression that her concerns were largely being ignored. When healthcare personnel finally called her home, she described their attitude as cold and “medical.” The team’s protective attire and scant verbal interactions with C. – while consulting frequently among themselves – all contributed to making her feel even more “psychologically distant” from the very people she was relying on to take care of her. C. began to believe that her worries were excessive and stopped “trying to make an emotional connection” with the health professionals, from whom she had initially expected a word of comfort. In the weeks following the in-home meeting, one of the team phoned her regularly once a week, but kept the conversation firmly focused on her observation of the confinement rules and on recording “objective parameters” (temperature, presence of breathing difficulties, etc.). C. shut down during these calls and only spoke to answer the questions put to her by the health worker. She began to think that her worries were “wrong” and unworthy of attention.
Although she was developing an increasingly judgmental attitude toward herself, she also progressively felt the need to share what had happened with someone who would really listen to her. Her husband was not a candidate given that he tended to minimize and trivialize her emotional concerns.
When she first met one of the authors for a consultation, her sense of being emotionally incompetent was evident. She stated that “normal people react differently,” while she herself “couldn’t stop crying during the lockdown, like a kid.” At first, she was unable to connect the extraordinary nature of the disruption to her everyday routines with the emotional reactions that had followed. The habitual meanings underpinning her self-assessments (“You’re just a country girl,” “You don’t know how the world works,” “You must be wrong to feel or think this,” and “Stop crying and complaining”) continued to prevail over any objective evaluation of the situation. The aim of the initial sessions was to listen closely and nonjudgmentally to her account and help her to link her emotional reactions to what had actually happened: drawing out this connection was intended to normalize her experiences, making them more understandable and acceptable to her (see the *normalization criterion*). This required the clinician to adopt a widened perspective that encompassed the personal, familial, cultural, and social contexts in which the sources of the client’s distress had come into play. In light of these contextual features, the clinician guided the client toward developing a more comprehensive understanding of her life conditions. He did so by encouraging the client to view her thoughts and emotions as reactions to (internal and external) stimuli, rather than as manifestations of an inner and immutable deficit (see the *criterion of widening the observational field*). The practitioner also sought to enhance the client’s awareness that these reactions had been strongly influenced by her personal history by fostering the construction of an alternative narrative framework, and specifically the view that the client had attempted to cope with an unprecedented series of unpredictable events based on the personal resources she felt she could count on. Such resources were deeply rooted in her own proximal and distal history and profoundly influenced the ways available to her for dealing with the events of the pandemic: the clinician noted that she could not have reacted any differently, given the exceptional nature of the events and the (personal, psychological, familial, social) resources at her disposal. To this end, he made connections between C.’s history and her present concerns. The clinician also made extensive use of a metaphorical analogy, drawing from the client’s personal history. Because the client’s mother had been bitterly critical toward her and excessively demanding considering her daughter’s age (“an inflexible schoolmistress,” in the shared language of the therapy), the client was continuously at risk of adopting the same attitude toward herself, and focusing exclusively on performance (“You should have done better!”) rather than taking into account the unfavorable conditions she had been obliged to face (“I could not have behaved or thought differently, considering that…”). This negotiated and reiterated metaphor helped the client to develop a strong sense of kindness toward the much-criticized little girl that she had been and to transfer this to a considerable extent to her “adult-self.” This, in turn, fostered the development of a nonjudgmental, positive attitude toward herself and her emotional life more generally, helping her to make new sense of the difficulties she had encountered in the process of coping with the pandemic (see the *criterion of fostering meaning-making processes through the use of narratives and metaphors*).

The second vignette, while also confirming the importance of contextual factors, touches more specifically upon the role of community resilience and the mitigating effects of experiencing connectedness and transcendence.

F., an only child, is a 39-year-old single woman who has been living in Milan for the past 10 years. Her family is wealthy and highly cultured; she herself is very bright and has broad cultural interests. Her parents have been always emotionally unreliable: in her everyday interactions with them, they display an unpredictable mix of support, indifference, and offensiveness. She attended university in another town, where she felt unable to live up to her parents’ expectation that she should be independent. During this troubled university period, she began taking drugs and abusing alcohol. A few years later, after hitting a particularly low point, she decided to give up drugs, and did so successfully and on her own. After completing her studies, she planned to pursue her longstanding dream of working abroad. However, the sudden news that her mother was seriously ill interrupted this project, and she decided not to leave so she could care for her mother. In recent years, she has worked on and off and decided to enroll at university again, although she feels like “a fish out of water.” Nevertheless, she has recently experienced a “pleasant sense of continuity” from devoting herself to work and recreational activities that “at last have meaning.”
The COVID-19 home confinement hit F. hard. On the one hand, she became increasingly concerned about the social and personal effects of the pandemic, worries that were exacerbated by news stories in the media, which she followed incessantly during the lockdown period. On the other hand, her sense of disconnect – which habitually took the form of feeling “unusual” or “odd” (on account of still attending university in her late thirties, being able to afford not to have a proper job, having unusual and varied cultural interests, etc.) – was progressively exacerbated by a lack of interaction with others due to the home confinement measures. Her sense of personal identity gradually became more fragile, in the absence of habitual reference points such as interpersonal interactions, daily routines, work, and recreational activities. She felt that the continuity in her life – which she had finally achieved after “meaningless years without building anything significant” – was in danger: the temporary disruption of her routines had triggered a sense of meaninglessness, associated with low mood, hypersomnia/insomnia, hypervigilance, and occasional convulsive crying.
F. found unexpected relief when – by chance – she tuned into an independent radio station, whose contents she perceived as surprisingly in harmony with her own worldview and interests. Day after day, F. felt increasingly more connected, reporting the clear perception that “there are people out there that I feel connected with: I’m not alone anymore!” She perceived this connection to be profound, rooted in the arts, human values, and cultural interests. Her contact and interactions with the radio presenters and their guests – although mediated by technology – had the effect of “turning something around.” First, the distress F. reported having experienced gradually remitted; second, she became determined to stop delaying completion of her university course and decided to seriously devote herself to her studies. In view of the above, F. requested a psychological consultation.
During the first session with the clinician, F. displayed a generally disparaging attitude toward herself, behaving as though she expected the clinician to judge her for being “odd” and “unusual.” The clinician challenged this “given” definition of herself, asking the client to explain what she meant by “odd” or “unusual.” Their ongoing exchanges on this topic progressively led the client to acknowledge that her “isolation made her feel like she was ‘unique,’ in the negative sense of the word”. She also recognized that she might have been feeling like this because she had hardly ever had the opportunity to meet people with similar interests and cultural awareness before tuning into the radio station (see the *criterion of normalization*). This discussion with the practitioner encouraged the client to do a “reality check” (as she evocatively said) and to “come out of my own narrow subjective perspective” (cit.) to consider the possibility that her personal experience – although private – could, nonetheless, be shared with many other individuals, at least on the basis of “our common human nature” (see the *criterion of widening the observational field*). This gradually led F. to develop a more appreciative attitude toward herself, as well as toward her own areas of knowledge, interests, and personal resources, in general. The practitioner pointed out to her that these resources can be useful for coping with reality, even in the face of an unprecedented event such as the pandemic: he connected the challenges of the present time with her skills and capacities. These comments were received with a mixture of suspicion and curiosity, and progressively fostered the construction of new meanings related to the events surrounding the pandemic. F. began to perceive herself differently, as “more resilient,” and found the courage to leave her home to safely meet other people. Borrowed from a popular saying, a metaphor emerged in the course of therapy, thus formulated by F.: “we are all experiencing the same storm. We are not in the same boat though: some people are sailing in fancy yachts with all the comforts, others in flimsy cheap rowing boats. Everyone is reacting in their own way to this pandemic, and there’s nothing wrong with that. I know I have to take care of my boat, the same as everyone else. Still, F., don’t forget there’s a storm out there!” (see the *criterion of fostering meaning-making processes through the use of narratives and metaphors*). This metaphor expressed F.’s gradually developing awareness of being personally responsible for her life decisions.

## Concluding Remarks

Undoubtedly, the dramatic impact of the COVID-19 pandemic on the mental health of the general population gives much cause for concern. Individuals, families, and communities have been cast back on their own resources on an unprecedented scale. In light of this scenario, we have attempted to sketch out a perspective on psychological intervention that we believe to be appropriate for addressing the crisis and its aftermath.

In summary, clinical interventions aimed at relieving the distressful consequences of the pandemic should fulfill a set of general, but crucial, criteria:

1. *Flexibility*. There should be scope for interventions to address forms of distress whose manifestations are blurred and/or milder than expected, and do not necessarily fully conform to any specific diagnosis.
2. *Personalization*. Interventions should be informed by an assessment of symptoms that takes the client’s underlying meaning systems into account, given that similar adverse stimuli may hold very different meanings for different individuals and in different contexts. Attending to the dimension of meaning should not exclude the use of symptom-centered techniques where required.
3. *Extended focus*. Interventions should conceptually link the development of mental problems to external triggers and stressors ascribable to the interpersonal domain (vs. solely internal triggers ascribable to the internal and/or biological domain). This is what we have defined as widening the field of observation to situate events (such as the client’s complaints or symptoms) within their interpersonal contexts and promote an understanding of distressful experiences as strategies for coping with objective external challenges.
4. *Emphasis on positive resources*. Intervention should foster awareness of personal, familial, and social resources and their deployment to develop personalized coping strategies, in a shift away from exclusively deficit- and disorder-centered outlooks.

We believe that all practitioners, independently of their clinical orientations, may benefit from adopting these overarching criteria. Our recommendations are not in conflict with the theoretical and methodological specifics of the various clinical approaches. Rather, we advocate for a broader psychological perspective on forms of distress associated with the negative cascade of events that has ensued upon the COVID-19 outbreak, suggesting that they call for a departure from more classically disorder-oriented and deficit-centered outlooks. In short, our ultimate purpose is to contribute to the debate on how traditional forms of clinical intervention may best be adapted to the novel contextual factors being shaped by the pandemic.

## Data Availability Statement

The original contributions presented in the study are included in the article/supplementary material, further inquiries can be directed to the corresponding author.

## Ethics Statement

Written informed consent was obtained from the individual(s) for the publication of any potentially identifiable images or data included in this article.

## Author Contributions

The authors equally contributed to the writing of the article. Both authors contributed to the article and approved the submitted version.

## Conflict of Interest

The authors declare that the research was conducted in the absence of any commercial or financial relationships that could be construed as a potential conflict of interest.

## Footnotes

1 There is evidence that pandemics may also have positive effects, such as strengthening individual resilience, increasing levels of social cohesion, and fostering positive concern about mental health (Perrin et al., 2009; Pfefferbaum and North, 2020).

2 The disruption of previous routines and the need to adjust to new ones represent a major source of stress for 74% of American parents.

3 A variety of labels are used in the literature to indicate these practices: telemedicine applied to mental health, telepsychology, mediated services, Internet- and mobile-based intervention.

4 In the interests of epistemological clarity, let us here summarize our perspective on “symptoms” throughout this paper: Far from viewing them as mere “facts,” we conceptualize symptoms (and their diagnosis) as–at least in part–theoretical constructs. Indeed, “one of the major conclusions from the philosophical analysis of science after the demise of logical empiricism in the second half of the 20th century has been that there is no such thing as notions of ‘experience’, ‘fact’, ‘evidence’, and the like (including ‘symptom’), which are *not* theoretically informed. In the wake of the tradition begun by philosophers and historians of science such as Hanson and Kuhn, a common place of contemporary philosophy of science is that any piece of scientifically relevant ‘evidence’ is, in fact, *theory laden* (Hanson, 1958), that is to say, it is meaningful only when viewed as part of a theoretical framework” (Castiglioni and Laudisa, 2015, p. 4). Nevertheless, in light of the specific aims of this paper, we do not set out here to make a systematic critique of the traditional diagnostic approach.

5 We acknowledge that the weaknesses attributed to the biomedical model certainly extend beyond the boundaries of CBT and indeed concern many models of psychological intervention. However, we also note that most of the interventions offered during the pandemic–whether face-to-face or technologically mediated–have been based on CBT principles, given the core features of this approach (i.e., symptom centeredness, focus on rapid interventions, replicability of interventions, quantifiability of results, etc.). These features make the CBT model particularly apt to be chosen for intervention in emergency situations such as the COVID-19 pandemic.

6 By “constructivist paradigm” we mean a sort of broad theoretical “umbrella” encompassing a range of theories, which in, some cases, are different, and “rival” to each other (Kuhn, 1962-1970). Attempting to analyze the differences between the positions of individual scholars would mean initiating a huge and potentially never-ending epistemological debate. Such an analysis would fall far outside the scope of this paper. The present article, whose specific focus is the impact of the COVID-19 pandemic, is generally informed by the constructivist perspective. For each of the main points in our argument, we cite the authors whose work we consider most salient. All the scholars whose work we draw on may be viewed as belonging to the “extended family” of constructivism, in all its different varieties and with all its different nuances. This should be sufficient–for the specific purposes of the present work–to ensure the overall epistemological coherence of our proposed perspective, leaving the analysis of the different constructivist positions to other works (see for example, to limit the field to clinical psychology: quotations omitted for anonymous peer review; Neimeyer and Mahoney, 1995; Castiglioni and Faccio, 2010; Castiglioni, 2011). Importantly, the key constructs on which we have based our perspective display a degree of commonality. Specifically, they share the following common points, which may be viewed as alternative to the premises of the biomedical model: (a) a focus on the importance of meaning-making processes; (b) an emphasis on the “embeddedness” of such processes in social/relational contexts.

7 From a “general” constructivist standpoint, we might say that mental distress arises when one feels that one’s usual system of meanings has become useless, unavailable, or unsound for the purposes of making sense of one’s current situation. Hence, the existing meaning-making system is “interrupted” or “suspended” before it has been possible to elaborate a sufficiently satisfactory “alternative type of construction” (whether by extending/further modifying the old one or by replacing it with a new one): no viable sense making (whether personal or “social”) appears to be currently possible (Kelly, 1955; Feixas et al., 2009).

8 Within this theoretical framework, Moscovici examined the process whereby scientific theories are translated into, and permeate, “common-sense” knowledge.

9 W I I stands for “We” (referring to one’s relationships with others), “I” (individuality and subjectivity), “It” (referring both to the natural world and human technological and scientific understanding of it). Thriving refers to a state of flourishing, optimal functioning, and fulfillment across all levels of analysis and time frames (see https://www.wiithrive.com/home).

## References

[B1] American Psychiatric Association. (2013). *Diagnostic and Statistical Manual of Mental Disorder*, 5th Edn Arlington, VA: Author.

[B2] American Psychological Association (2020). *Stress in the Time of COVID-19.* Massachusetts, MA: American Psychological Association.

[B3] Andrade L. H. Alonso J. Menimneh Z. Wells J. E. Al-Hamzavi A. Borges G. (2014). Barriers to mental health treatment: results from the WHO World Mental Health (WMH) Surveys. *Psychol. Med.* 44 1303–1317. 10.1017/S0033291713001943 23931656 PMC4100460

[B4] Anglin D. M. Polanco-Roman L. (2017). “Deficit model,” in *The SAGE Encyclopedia of Abnormal and Clinical Psychology*, ed. Wenzel A. (London: Sage), 998–999. 10.4135/9781483365817.n381

[B5] Antonovsky A. (1979). *Health, Stress and Coping.* San Francisco, CA: Jossey-Bass.

[B6] Antonovsky A. (1987). *Unraveling the Mystery of Health.* San Francisco, CA: Jossey-Bass.

[B7] Antonovsky A. (1993). The structure and properties of the sense of coherence scale. *Soc. Sci. Med.* 36 725–733. 10.1016/0277-9536(93)90033-z 8480217

[B8] Antonovsky A. (1996). The salutogenic model as a theory to guide health promotion. *Heath Promot. Int.* 11 11–18. 10.1093/heapro/11.1.11

[B9] Ao B. (2020). *More People are Using Antidepressants and Antianxiety Medications during Coronavirus Pandemic: Survey.* Philadelphia, PA: The Philadelphia Inquirer.

[B10] Austin J. L. (1962). *in How to do things with Words*, eds Urmoson J. O. Sbisà M. (Harvard: Harvard University Press), 1975.

[B11] Bashshur R. L. Shannon G. W. Bashshur N. Yellowlees P. M. (2016). The empirical evidence for telemedicine interventions in mental disorders. *Telemed. E Health* 22 87–113. 10.1089/tmj.2015.0206 26624248 PMC4744872

[B12] Bateson G. (1972). *Steps to an Ecology of Mind.* San Francisco, CA: Chandler.

[B13] Battistelli F. (2020). *Guerra al Coronavirus, Prevenire è Meglio che Combattere.* Available online at: http://www.vita.it/it/article/2020/03/31/guerra-al-coronavirus-prevenire-e-meglio-che-combattere/154794/ (accessed May 28, 2020).

[B14] Bentall R. P. (2009). *Doctoring the Mind. Why Psychiatric Treatments Fail.* London: Penguin Books.

[B15] Bradford G. K. (2010). Fundamental flaws of the DSM: re-envisioning diagnosis. *J. Humanist. Psychol.* 50 335–350. 10.1177/0022167809352007

[B16] Breitbart W. Rosenfeld B. Gibson C. Pessin H. Poppito S. Nelson C. (2010). Meaning-centered psychotherapy for patient with advanced cancer: a pilot randomized controlled trial. *Psychooncology* 19 21–28. 10.1002/pon.1556 19274623 PMC3648880

[B17] Brooks S. K. Webster R. K. Smith L. E. Woodland L. Wessely S. Greenberg N. (2020). The psychological impact of quarantine and how to reduce it: rapid review of the evidence. *Lancet* 395 912–920. 10.1016/s0140-6736(20)30460-8 32112714 PMC7158942

[B18] Bruner J. S. (1990). *Acts of Meaning.* Harvard: Harvard University Press.

[B19] Bruner J. S. (1991). The narrative construction of reality. *Crit. Inq.* 18 1–21.

[B20] Castiglioni M. (2011). Departing from classical logic: a logical analysis of personal construct theory. *J. Constr. Psychol.* 24 93–121. 10.1080/10720537.2011.548210

[B21] Castiglioni M. Faccio E. (2010). *Costruttivismi in psicologia clinica. Teorie, metodi e ricerche.* Novara: Utet.

[B22] Castiglioni M. Laudisa F. (2015). Toward psychiatry as a ‘human’ science of mind. The case of depressive disorders in DSM-5. *Front. Psychol.* 5:1517. 10.3389/fpsyg.2014.01517 25601847 PMC4283446

[B23] Castiglioni M. Veronese G. Pepe A. Villegas M. (2014). The semantics of freedom in agoraphobic patients: an empirical study. *J. Constr. Psychol.* 27 120–136. 10.1080/10720537.2013.806874

[B24] Deacon B. J. (2013). The biomedical model of mental disorder: a critical analysis of its validity, utility, and effects on psychotherapy research. *Clin. Psychol. Rev.* 33 846–861. 10.1016/j.cpr.2012.09.007 23664634

[B25] Delgado C. (2007). Sense of coherence, spirituality, stress and quality of life in chronic illness. *J. Nurs. Scholarsh.* 39 229–234. 10.1111/j.1547-5069.2007.00173.x 17760795

[B26] Drageset J. Nygaard H. A. Eide G. E. Bondevik M. Nortvedt M. W. Natvig G. K. (2008). Sense of coherence as a resource in relation to health-related quality of life among mentally intact nursing home residents: a questionnaire study. *Health Q. Life Outcomes* 6:85. 10.1186/1477-7525-6-85 18940001 PMC2607268

[B27] Ebert D. D. Van Daele T. Nordgreen T. Karekla M. Compare A. Zarbo C. (2018). Internet- and Mobile-based psychological interventions: applications, efficacy, and potential for improving mental health. A report of the EFPA E-Health Taskforce. *Eur. Psychol.* 23 167–187. 10.1027/1016-9040/a000318

[B28] Eriksson M. Lindström B. (2005). Validity of Antonovsky’s sense of coherence scale: a systematic review. *J. Epidemiol. Commun. Health* 59 460–466. 10.1136/jech.2003 PMC1757043 15911640

[B29] Feixas G. Saúl L. A. Ávila-Espada A. (2009). Viewing cognitive conflicts as dilemmas: implications for mental health. *J. Constr. Psychol.* 22 141–169. 10.1080/10720530802675755 22629109 PMC3339575

[B30] Frances A. (2013). *Saving Normal.* New York, NY: Harper Collins Publishers.

[B31] Guidano V. F. (1991). *The Self in Process.* New York, NY: Guilford.

[B32] Gunnell D. Appleby L. Arensman E. Hawton K. John A. Kapur N. (2020). Suicide risk and prevention during the COVID-19 pandemic. *Lancet Psychiatry* 7 468–471. 10.1016/S2215-0366(20)30171-1 32330430 PMC7173821

[B33] Hanson R. H. (1958). *Patterns of Discovery: An Inquiry into the Conceptual Foundations of Science.* Cambridge: Cambridge University Press.

[B34] Henriques G. R. (2002). The harmful dysfunction analysis and the differentiation between mental disorder and disease. *Sci. Rev. Ment. Health Pract.* 1 157–173.

[B35] Herman J. (2015). *Trauma and Recovery. The Aftermath of Violence – from Domestic Abuse to Political Terror.* New York, NY: Basic Books.

[B36] Higgins T. (2020). *Coronavirus Pandemic could Inflict Emotional Trauma and PTSD on an Unprecedented Scale, Scientists Warn.* Englewood Cliffs, NJ: CNBC.

[B37] Holland J. M. Currier J. M. Coleman R. A. Neimeyer R. A. (2010). The integration of stressful life experiences scale (ISLES): development and initial validation of a new measure. *Int. J. Stress Manag.* 17 325–352. 10.1037/a0020892

[B38] Holmes E. A. O’Connor R. C. Perry V. H. Tracey I. Wessely S. Arseneault L. (2020). Multidisciplinary research priorities for the COVID-19 pandemic: a call for action for mental health science. *Lancet Psychiatry* 7 547–560. 32304649 10.1016/S2215-0366(20)30168-1 PMC7159850

[B39] Hooghe A. Neimeyer R. A. Rober P. (2012). Cycling around an emotional core of sadness. Emotion regulation in a couple after the loss of a child. *Q. Health Res.* 22 1220–1231. 10.1177/1049732312449209 22745365

[B40] Horwitz A. V. Wakefield J. C. (2007). *The Loss of Sadness: How Psychiatry Transformed Normal Sorrow into Depressive Disorder.* Oxford: Oxford University Press. 10.1176/appi.ajp.2007.07081263 22688233

[B41] Hubley S. Lynch S. B. Schneck C. Thomas M. Shore J. (2016). Review of key telepsychiatry outcomes. *World J. Psychiatry* 6 269–282. 10.5498/wjp.v6.i2.269 27354970 PMC4919267

[B42] Hucklenbroich P. (2017). “Medical theory and its notions of definitions and explanation,” in *Handbook of the Philosophy of Medicine*, eds Schramme T. Edwards S. (Dordrecht: Springer), 793–801. 10.1007/978-94-017-8688-1_44

[B43] Hunt N. C. (2010). *Memory, War and Trauma.* Cambridge: Cambridge University Press.

[B44] Jacobs D. H. Cohen D. (2010). Does “psychological dysfunction” mean anything? A critical essay on pathology versus agency. *J. Humanist. Psychol.* 50 312–334. 10.1177/0022167809352008

[B45] Jacobs K. A. (2013). The depressive situation. *Front. Psychol.* 4:429. 10.3389/fpsyg.2013.00429 23882238 PMC3713339

[B46] Johnstone L. Boyle M. (2018). *The Power Threat Meaning Framework: Towards the Identification of Patterns in Emotional Distress, Unusual Experiences and Troubled or Troubling Behavior, as an Alternative to Functional Psychiatric Diagnosis.* Leicester: British Psychological Society.

[B47] Kelly G. A. (1955). *). The Psychology of Personal Constructs.* New York, NY: Norton.

[B48] Kimhi S. Eshel Y. Zysberg L. Hantman S. Enosh G. (2010). Sense of coherence and sociodemographic characteristics predicting posttraumatic stress symptoms and recovery in the aftermath of the second Lebanon war. *Anxiety Stress Coping An Int. J.* 23 139–152. 10.1080/10615800902971513"> 19452309

[B49] Kuhn T. S. (1962–1970). *The Structure of Scientific Revolutions.* Chicago: The University of Chicago Press.

[B50] Lund K. Argentzell E. Leufstadius C. Tjörnstrand C. Eklund M. (2017). Joining, belongingand re-valuing: a process of meaning-making through group participation in a mental health lifestyle intervention. *Scand. J. Occup. Ther.* 26 55–68. 10.1080/11038128.2017.1409266 29179630

[B51] Milesi S. (2020). *La Viralità Del Linguaggio Bellico.* Available online at: http://www.vita.it/it/article/2020/03/26/la-viralita-del-linguaggio-bellico/154699/ (accessed May 27, 2020).

[B52] Moscovici S. Poloma M. Hood J. R. (2001). *Social Representations: Essays in Social Psychology.* New York, NY: New York University Press.

[B53] Neimeyer R. A. (2009). *Constructivist Psychotherapy.* London: Routledge.

[B54] Neimeyer R. A. Mahoney M. J. (eds) (1995). *Constructivism in Psychotherapy.* Washington, DC: American Psychological Association.

[B55] Neimeyer R. A. Sands D. C. (2011). “Meaning reconstruction in bereavement. from principles to practice,” in *Grief and Bereavement in Contemporary Society. Bridging Research and Practice*, eds Neimeyer R. A. Harris D. Winokuer H. Thornton G. (New York, NY: Routledge), 34–47.

[B56] Neimeyer R. A. Harris D. L. Winokuer H. R. Thornton G. F. (2011). *Grief and Bereavement in Contemporary Society. Bridging Research and Practice.* London: Routledge.

[B57] Neimeyer R. A. Klass D. Dennis M. R. (2014). A social constructionist account of grief: Loss and the narration of meaning. *Death Stud.* 38 485–498. 10.1080/07481187.2014.913454 24738824

[B58] Nguyen-Gillham V. Giacaman R. Naser G. Boyce W. (2008). Normalising the abnormal: Palestinian youth and the contradictions of resilience in protracted conflict. *Health Soc. Care Community* 16 291–298. 10.1111/j.1365-2524.2008.00767.x 18355248

[B59] Perrin P. C. McCabe O. L. Everly G. S. Links J. M. (2009). Preparing for an influenza pandemic: Mental health considerations. *Prehosp. Disast. Med.* 24 223–230. 10.1017/S1049023X00006853 19618359

[B60] Pesce N. L. (2020). *Anti-Anxiety Medication Prescriptions have Spiked 34% During the Coronavirus Pandemic.* New York, NY: Market Watch.

[B61] Pfefferbaum B. North C. S. (2020). Mental health and the COVID-19 pandemic. *N. Engl. J. Med.* 383 510–512. 10.1056/NEJMp2008017 32283003

[B62] Polkinghorne D. E. (2004). “Narrative therapy and postmodernism,” in *The Handbook of Narrative and Psychotherapy: Practice, Theory and Research*, eds Angus L. E. McLeod J. (London: Sage), 52–67. 10.4135/9781412973496.d5

[B63] Prime H. Wade M. Browne D. T. (2020). Risk and Resilience in Family Well-Being During the COVID-19 Pandemic. *Am. Psychol.* 75 631–643. 10.1037/amp0000660 32437181

[B64] Procaccia R. Neimeyer R. A. Veronese G. Castiglioni M. (2018). Children’s representations of death: the role of age and attachment style. *TPM Test. Psychom. Methodol. Appl. Psychol.* 25 549–569. 10.4473/TPM25.4.6

[B65] Raskin J. D. Bridges S. K. (eds) (2002). *Studies in Meaning: Exploring Constructivist Psychology.* New York, NY: Pace University Press.

[B66] Razer M. Friedman V. J. Veronese G. (2009). The “educational–psychosocial approach” to overcoming social exclusion in Israeli schools. *Ricerche Di Psicol.* 32 219–235.

[B67] Sagy S. Antonovsky H. (2000). The development of the sense of coherence: a retrospective study of early life experiences in the family. *J. Aging Hum. Dev.* 51 155–166. 10.2190/765l-k6nv-jk52-ufkt 11140850

[B68] Schimmenti A. Billieux J. Starcevic V. (2020). The four horsemen of fear: an integrated model of understanding fear experiences during the COVID-19 pandemic. *Clin. Neuropsychiatry* 17 41–45. 10.36131/CN20200202 PMC8629088 34908966

[B69] Seligman M. E. P. Steen T. A. Park N. Peterson C. (2005). Positive psychology progress: empirical validation of interventions. *Am. Psychol.* 60 410–421. 10.1037/0003-066X.60.5.410 16045394

[B70] Shore J. H. Schneck C. D. Mishkind M. C. (2020). Telepsychiatry and the coronavirus disease 2019 pandemic – Current and future outcomes of the rapid virtualization of psychiatric care. *JAMA Psychiatry* 10.1001/jamapsychiatry.2020.1643 Online ahead of print 32391861

[B71] Shotter J. (1993). *Cultural Politics of Everyday Life.* Milton Keynes: Open University Press.

[B72] Slife B. D. Martin G. Sasser S. (2017). “A prominent worldview of professional psychology,” in *The Hidden Worldviews of Psychological Theory, Research, And Practice*, eds O’Grady K. A. Kosits R. D. (New York, NY: Routledge), 25–43. 10.4324/9781315283975-3

[B73] Smith L. Romero L. (2010). Psychological interventions in the context of poverty: Participatory action research as practice. *Am. J. Orthopsychiatry* 80 12–25. 10.1111/j.1939-0025.2010.01003.x 20397985

[B74] Sood S. (2020). Psychological effects of the coronavirus COVID-2019 pandemic. *RHiME* 7 23–26.

[B75] Squire C. Andrews M. Davis M. Esin C. Harrison B. Hyden L. C. (2014). *What is Narrative Research?*, 2nd Edn London: Bloomsbury.

[B76] VandenBos G. R. (2015). *APA Dictionary of Psychology.* 2nd Edn Washington, DC: American Psychological Association.

[B77] Venuleo C. Gelo C. G. O. Salvatore S. (2020). Fear, affective semiosis, and management of the pandemic crisis: COVID-19 as semiotic vaccine? *Clin. Neuropsychiatry* 17 117–130. 10.36131/CN20200218 PMC8629038 34908982

[B78] Veronese G. Castiglioni M. Barola G. Said M. (2012). Living in the shadow of occupation: life satisfaction and positive emotion as protective factors in a group of Palestinian school children. *Child Youth Serv. Rev.* 34 225–233. 10.1016/j.childyouth.2011.10.002

[B79] Veronese G. Castiglioni M. Said M. (2010). The use of narrative experiential instruments in contexts of military violence: the case of Palestinian children in the West Bank. *Couns. Psychol. Q.* 23 411–423. 10.1080/09515070.2010.529678

[B80] Veronese G. Fiore F. Castiglioni M. Al-Kawaja H. Said M. (2013). Can sense of coherence moderate traumatic reactions? a cross-sectional study of palestinian helpers operating in war contexts. *Br. J. Soc. Work* 43 651–666. 10.1093/bjsw/bcs005

[B81] Walsh F. (1998). “Beliefs, spirituality, and transcendence: Keys to family resilience,” in *Re-Visioning Family Therapy: Race, Culture, and Gender in Clinical Practice*, ed. Mc Goldrick M. (New York, NY: Guilford), 62–77.

[B82] Walsh F. (2010). *Spiritual Resources in Family Therapy*, 2nd Edn New York, NY: Guilford.

[B83] Walsh F. (2015). *Strengthening Family Resilience.* New York, NY: Guilford.

[B84] Watzlawick P. Beavin J. H. Jackson D. D. (1967). *Pragmatics of Human Communication.* New York, NY: Norton.

[B85] World Health Organization. (2018). *International Classification of Diseases for Mortality and Morbidity Statistics (11th Revision).* Available online at: https://icd.who.int/browse11/l-m/en (accessed June 5, 2020).

[B86] World Health Organization (2020). *Mental Health and Psychosocial Considerations during the COVID-19 Outbreak.* Geneva: World Health Organization.

[B87] Yalom I. D. Leszcz M. (2005). *The Theory and Practice of Group Psychotherapy*, 5th Edn New York, NY: Basic Books.

